# Longitudinal associations between self-regulation and physical activity behavior following metabolic bariatric surgery; an exploratory study

**DOI:** 10.1186/s12966-025-01739-2

**Published:** 2025-04-08

**Authors:** C. Sundgot-Borgen, S. Baardstu, D. S. Bond, F. F. Sniehotta, I. Bergh, T. Mala, Ø. Rø, I. L. Kvalem

**Affiliations:** 1 Regional Department for Eating Disorders, Division of Mental Health and Addiction, Oslo University Hospital, 4956, Nydalen Oslo, 0424 Norway; 2 Department of Psychology, University of Oslo, 1094, Blindern Oslo, 0317 Norway; 3 Department of Childhood and Families, the Norwegian Institute of Public Health, 222, Skøyen Oslo, 0213 Norway; 4 Center for Obesity Research, Innovation and Education, Digestive Health Institute, Hartford Healthcare, Hartford, CT 06102 USA; 5 Centre for Preventive Medicine and Digital Health, Division Public Health, Preventive and Social Medicine, Medical Faculty Mannheim, Heidelberg University, Röntgenstraße 7, D- 68167 Mannheim, Germany; 6 Nudgelab, Konghellegata 10, 0570 Oslo, Norway; 7 Center for Morbid Obesity and Bariatric Surgery, Oslo University Hospital, 4950, Nydalen, 0424 Norway; 8 Institute of Clinical Medicine, University of Oslo, 1171, Blindern Oslo, 0318 Norway; 9 Institute of Clinical Medicine, University of Oslo, 1039, Blindern Oslo, 0315 Norway

**Keywords:** Metabolic bariatric surgery, Self-regulation, Physical activity, Exercise, Lifestyle

## Abstract

**Background:**

Low adherence to moderate-to-vigorous physical activity (MVPA) recommendations among patients undergoing metabolic bariatric surgery (MBS) is common. However, understanding of psychosocial factors that contribute to low adherence levels is limited. Self-regulation plays a key role in MVPA adherence. Still, the longitudinal and bidirectional associations between self-regulation and MVPA in the MBS patient population remains unexplored. This study aimed to investigate how self-regulatory processes of action- and coping planning, and action control, developed over a 1–5-year post-surgery period, and explore longitudinally the direct, indirect, and bidirectional associations between this development in self-regulation and MVPA assessed at 1- and 5-years after surgery.

**Methods:**

Participants from the Oslo Bariatric Surgery Study (OBSS) completed MVPA-specific self-regulation questionnaires at 1-, 3-, and 5-years post-surgery and wore ActiGraph monitors for seven days at 1- and 5-years to assess daily MVPA. Second-order latent growth curve modeling examined changes in the three self-regulation constructs, followed by path analysis to explore direct, indirect, and bidirectional associations between baseline levels and changes in self-regulation, and MVPA at 1- and 5-years post-surgery.

**Results:**

A total of 205 (82.8%), 195 (64.6%), and 79 (26.2%) male and females (77%) participated at 1-, 3-, and 5-years after surgery, respectively. Action- and coping- planning decreased with.52 and.30 sd, respectively, over 1–5 years post-surgery. This indicates a moderate effect size. Action control remained relatively stable. Indirect and bidirectional path analyses showed that only higher levels of action control at 1-year were indirectly associated with higher MVPA at 5-years through their impact on MVPA at 1-year, whereas there were no indications of bidirectional associations from MVPA levels at 1-year to changes in any of the self-regulation constructs over time.

**Conclusions:**

After MBS, patients exhibited low self-regulation 1-year post-surgery, and many participants faced growing difficulties in self-regulating over time. Self-regulation at 1-year was positively linked to MVPA, with action control only, being associated with MVPA at 5-years. However, no bidirectional associations from MVPA to self-regulation were found. These findings suggest further research is needed to test interventions targeting action control to improve MVPA adherence and optimize surgical outcomes and overall health after MBS.

**Supplementary Information:**

The online version contains supplementary material available at 10.1186/s12966-025-01739-2.

## Background

To minimize the amount of weight recurrence and improve overall physical and mental health, metabolic bariatric surgery (MBS) patients are advised to follow the public health guideline of ≥ 150 min of moderate-to-vigorous intensity physical activity (MVPA) per week [1–3]. Objectively-measured MVPA levels are typically low before surgery [4–8], and although longitudinal studies show variability in direction and magnitude of MVPA changes after surgery, findings are consistent in showing that post-surgical MVPA levels often fall short of MVPA recommendations [4, 9–13]. Similar findings were shown in a previous study from the Oslo Bariatric Surgery Study (OBSS), in which a low percentage of patients met MVPA guidelines 1- (23.3%) and 5-years (20.5%) after surgery [14]. This gap between current recommendations and patients’ MVPA level is important because lower MVPA levels are associated with more weight recurrence [4, 15–21] and poorer overall long-term health after MBS [19, 20].

Given that most patients do not make clinically meaningful changes in MVPA after MBS, despite experiencing substantial weight loss and health improvements, there is a need to better understand how psychosocial factors influence MVPA adherence and vice-versa after MBS. One important psychosocial factor is self-regulation.

Self-regulation is an individual’s capacity to alter behavior, which involves gaining control over thoughts, emotions, behavior, and attention to reach a specific behavior goal such as meeting MVPA recommendation [22]. Although robust evidence show that intention (the motivation for, and the specific decisions to be physically active) and self-efficacy (a person’s beliefs and confidence in one’s abilities to perform the intended physical activity (PA)) strongly associates with MVPA levels [23–27], the intention to- and confidence in-, becoming more physically active does not necessarily translate into sustained action. This phenomenon is also known as the “intention-behavior gap” [28], and can be attributed to factors such as inadequate planning and struggle with initiating and maintaining the intended action (action control) [28–30].

Planning how to change behavior (action planning) and how to cope with difficult situations in order to uphold the plans (coping planning) [31, 32] have been found to mediate the relationship between intention and MVPA behavior [33, 34]. Although given little attention, action control (entails self-monitoring, awareness of standards, and efforts to reduce discrepancies between one`s standards and one`s current behavior), has been described as essential in terms of helping to translate intention into action and maintain action over time [32, 35, 36].

There is to our knowledge, no existing evidence on prospective associations between self-regulation and MVPA in the context of MBS. However, higher levels of intention and self-efficacy has been found to associate with more MVPA [37, 38], and that action control potentially plays a key role in meeting MVPA recommendations [38, 39], and in the change of MVPA level after surgery [39, 40]. No studies have examined the relationship between self-regulation and MVPA beyond the immediate postoperative period, when MVPA adherence may be most important for preventing long-term weight recurrence [7, 38–41]. Because MBS patients have difficulty adopting and sustaining MVPA, there is a need for better understanding of how planning and action control are associated with MVPA after surgery, and especially how such associations may change over time.

The current study is therefore novel in its design and focus and will contribute to fill this knowledge gap by investigating whether planning and action control changes over time after surgery and how this relates to MVPA level in MBS patients. Most importantly, this knowledge could contribute to the development of behavioral interventions to achieve sustainable increases in MVPA and long-term adherence to MVPA guidelines.

## Methods

### Study aims

This study aimed to 1) investigate how PA-specific action planning, coping planning, and action control developed (i.e., changed) from 1-, 3-, and 5-years after surgery and, 2) explore longitudinally the direct, indirect, and bidirectional associations between this development and levels of MVPA assessed at 1- and 5-years after surgery.

### Participant characteristics and study design

The OBSS [42, 43] is a prospective cohort study of patients admitted to MBS, either sleeve gastrectomy or Roux-en-Y gastric bypass, at the Centre for Morbid Obesity and Bariatric Surgery at Oslo University Hospital, Norway, from 2011 to 2013. The 1991 National Institutes of Health Consensus Development Conference Statement for indication of MBS were applied throughout the study period. Clinical follow-up consultations were scheduled at 6 months, 1-, 3-, and 5-years after surgery. Volunteers to the OBSS were asked to respond to a questionnaire prior to surgery, 1-, 3-, and 5-years after surgery. Objectively measured MVPA was first investigated at 1- year after surgery, in which study findings have previously been reported on [38].

Recruitment of OBSS participants for MVPA evaluations 1-year after surgery has been described previously [44]. All participants who accepted to wear an ActiGraph at 1-year and returned valid data (N = 112) were eligible to take part in the 5-year follow-up study. Participants were asked to wear an ActiGraph GT3X + accelerometer for seven consecutive days. Delays in invitation to the 5-years ActiGraph data collection resulted in participants being invited to wear the monitor 7.88 (sd: 0.75) years after surgery. The 5-year data were collected in 2020, during the COVID- 19 pandemic.

**Fig. 1:**
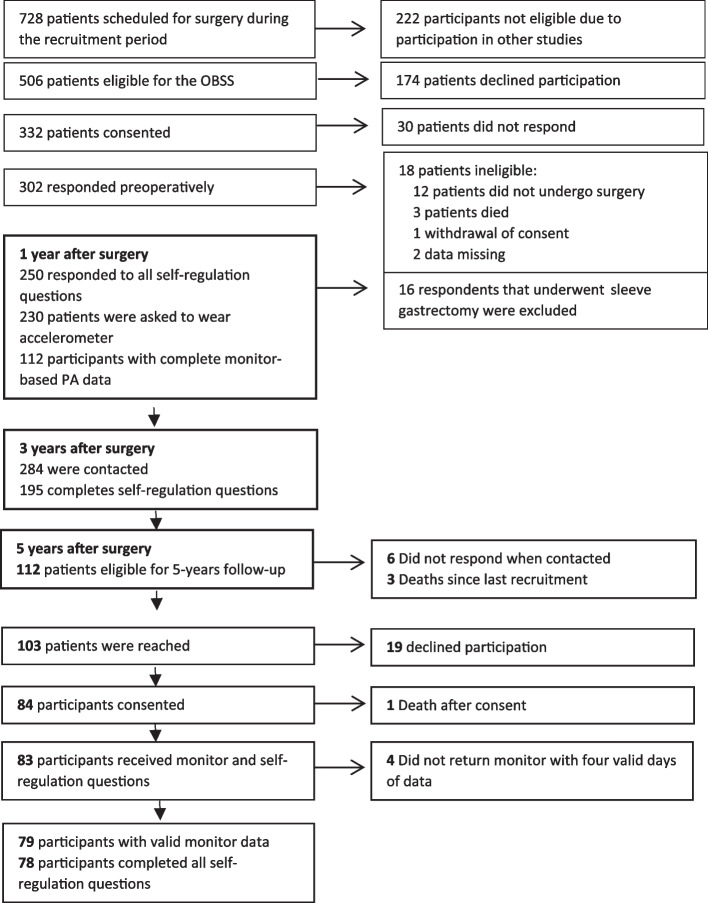
Participation flow

PA-specific self-regulation data on action- and coping planning and action control were reported at 1-, 3-, and 5-years after surgery. In contrast to other assessment time-points, only participants who took part in the objective assessment of MVPA at 5-years, were asked to respond to questions about PA-specific self-regulation at 5-years after surgery (Fig. 1). Data that were used in the present study are limited to those from the 1-year, 3-year, and 5-year follow-up periods.

### Measures

#### Self-regulation

Self-regulation was measured by previously validated PA-specific scales for action planning (5-items), coping planning (4-items) [28], and action control (6-items including self-monitoring, awareness of standards and efforts) [32], with respondents reporting on a Likert-scale ranging from 1–4 (Strongly disagree – Strongly agree) (See Additional File 1) [32].

#### Objectively measured MVPA

The ActiGraph GT3X + activity monitor (ActiGraph, LLC, Pensacola, FL, USA) was used to measure MVPA. The participants wore the accelerometers on their right hip during all waking hours for seven consecutive days, except during showering and bathing. ActiGraph data was included into analyses if containing at least four days with more than 10 h of valid data per day [45]. The accelerometer data was used to assess total minutes in MVPA per day calculated from mean counts per minute (cpm), moderate- (2020–5998 cpm) to vigorous intensity (≥ 5999 cpm) [45, 46]. When analyzing data, total minutes in MVPA were used due to low accumulation of bouted MVPA minutes in our sample [14]. This is however, also in accordance with the 2018 Physical Activity Guidelines Advisory Committee [47], and 2020 guidelines recommending a weekly MVPA of at least 150 min [2].

#### Weight

Weight was measured using a calibrated Seca 635 III (0–300 kg) platform scale at follow-up consultations at 1-, 3-, and 5-years after surgery, with light clothing and no shoes. For eight participants, self-reported weight replaced lack of objective measurements and was considered valid due to high correlation between subjectively and objectively measured weight pre-surgery (r = 0.96, *p* = < 0.001). Total weight loss was calculated as %TWL = [(Weight on the day of surgery) – (Postoperative Weight)]/(Weight on the day of surgery) × 100 [48], and further categorized as ≥ 20% and < 20% of pre-surgery weight [49]. Nadir weight was defined as the lowest postoperative body weight objectively assessed at 1-, 3- and 5-years after surgery. Weight recurrence was used as a continuous and dichotomous variable, and calculated by 100*(Weight at 5-years follow-up—nadir)/(pre-surgery weight—nadir), and dichotomized into ≥ 20% or < 20% of maximum weight loss [50]. Body mass index (kg/m^2^) (BMI) was calculated at the day of surgery and at medical follow-up consultations at 1-, 3-, and 5-years after surgery.

Patients self-reported age, employment status, educational level, whether they had a partner or were married, and whether they were cohabitants.

### Statistical analyses

Preliminary and descriptive analyses were performed using IBM SPSS version 26 and the main analyses were conducted in the framework of structural equation modeling (SEM) using Mplus version 8.9 [51]. To handle missing data, full information maximum likelihood estimation was used.

To evaluate differences between those who completed self-regulation questionnaires at 3-years vs. participants not completing the follow-ups, independent sample t-test were used for continuous variables (body weight, mean score in all self-regulation sub-scales, and mean total minutes in MVPA per day) and chi-square for categorical data (education, relationship status, employment status, gender), at 1-year after surgery. Almost all participants at 5-years completed all self-regulation questions, and similar analyses were not appropriate for this time-point. Independent sample t-tests were used to evaluate differences in age, weight loss, weight, and MVPA, at 1-year after surgery, between those who completed MVPA assessment at both time-points and dropouts from 1- to 5-years follow-up. Two-tailed *p*-values at ≤ 0.05 were evaluated significant. To evaluate correlations between self-regulation and MVPA at each time-point, Pearsons’s correlation was used.

The main analyses were performed in several steps. First, confirmatory factor analyses (CFA) were performed to construct latent factors for the subscales action control, action planning, and coping planning for each time-point based on their respective items.

Second, as measurement invariance is an important requirement for longitudinal analyses, tests of factorial invariance across the three time-points were then performed for each of the latent factors by comparing a baseline model (i.e., configural invariance) with a series of increasingly restricted models (i.e., weak and strong invariance), following Widaman, Ferrer, and Conger [52]. Goodness of fit comparisons between the invariance models were evaluated by using the χ^2^ statistic, the root mean square error of approximation (RMSEA), the comparative fit index (CFI), and the Tucker-Lewis Index (TLI), following the cut-off criteria specified by Hu and Bentler [53] (See Supplementary Table 1, Additional File 2).

Third, development over time for the action control, action planning and coping planning constructs was tested by constructing second-order latent growth curve models for each construct based on their latent factors at each time-point. Two growth parameters were estimated for each construct, 1) the intercept, representing the estimated baseline (i.e., starting) levels of the constructs at 1-year, and 2), the slope, representing the average rate of change across the three measurements.

**Fig. 2:**
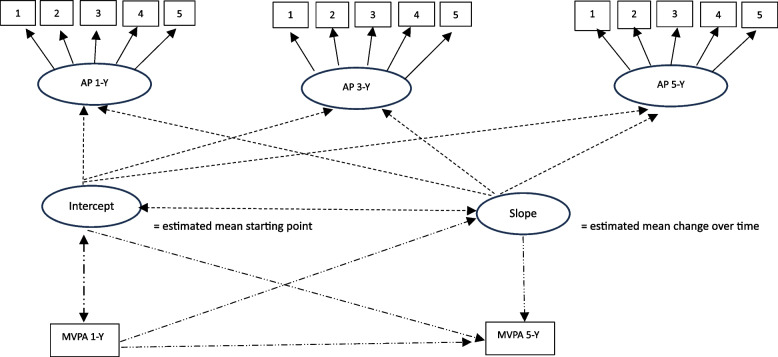
Visualization of the growth curve model and of the structural model. Visualization of the measurement model (i.e., growth curve model) of the development of self-regulation subscales over time (here action planning is used as an example) and of the structural model testing direct, indirect, and bidirectional associations with MVPA over time. Solid arrows = Confirmatory factor analyses (CFA). Short, dotted arrows = growth curve analysis. Long dotted arrows = correlation and regression analyses, including direct, indirect, and bidirectional effects. AP = action planning. MVPA = moderate to vigorous physical activity, Y = year

Fourth, to test the direct, indirect, and bidirectional associations between the latent action control, action planning and coping planning factors and the objective measure of MVPA at 1- and 5-years after surgery, a series of regression models were performed. First, we estimated a regression model for each of the three subscales using path analyses. In each of these models, the MVPA measure at 1-year and the intercept and slope factors of the self-regulation construct were specified as predictors of the MVPA measure at 5-years while the intercept was specified as predictor of MVPA at 1-year (See Fig. 2). Here, we also estimated the indirect paths from the intercepts to MVPA at 5-years through MVPA at 1-year as well as testing bidirectional paths from MVPA at 1-year to the growth factors (i.e., both the intercept and the slope) of each of the subscales over time by using bootstrapping with bias-corrected confidence intervals [54]. Then, to further probe which aspect of self-regulation that would have the strongest association with MVPA, we also specified a model where all growth curve models for all the three subscales were estimated simultaneously and where the intercepts (i) and slopes (s) for all the subscale factors were specified as predictors of MVPA at 5-years (i1 and s1 for action control, i2 and s2 for coping planning, and i3 and s3 for action planning), and where all intercepts were specified as predictors of MVPA at 1-year. Also, for this model, we estimated indirect and bidirectional paths from the intercepts to MVPA at 5-years through MVPA at 1-year and from MVPA at 1-year to the intercepts and slopes of the self-regulation factors, using bootstrapping with bias-corrected confidence intervals. Total percentage of weight loss and cohabitation status at 1-year were included as covariates in all regression models.

## Results

Based on the 302 participants who responded after surgery, the response rate for self-regulation questions was 82.8%, 64.68.7%, and 26.2 at 1-, 3-, and 5-years after surgery, respectively. At 1-, and 5-years, 49% and 70% of participants who received the monitor to wear, returned valid ActiGraph data, respectively (See Fig. 1).

Participants who only completed self-regulation questions at 1-year, and not at 3-years, performed less MVPA compared to completers at both time-points (mean diff: − 8.92 min, 95%CI: 2.27, 15.56, *p* = 0.009). Participants who took part in the MVPA monitoring at both time-points had a lower body weight at 1-year follow-up compared to dropouts (mean diff: − 13.29 kg, 95%CI: − 22.19, − 4.39, *p* = 0.002). (Characteristics of completers at 1-year who were eligible for 5-years follow-up assessment (N = 112) are presented in Supplementary Table 2 additional file 3.)

Participant characteristics at baseline and during follow-up are described in Table 1. The proportion of participants with normal weight and obesity seemed to decline and increase, respectively. At 5-years, more than half of the participants had regained more than 20% of maximum weight loss (Table 1).

**Table 1:** Description of participants in the Oslo Bariatric Surgery Study at each assessment time-point after surgery

	**Pre-surgery**			**1-y**			**3-y**			**5-y**		
	N	Mean/sum	Sd/%	N	Mean/ sum	Sd/%	N	Mean/ sum	Sd/%	N	Mean/ sum	Sd/%
Age	288	43.9	9.6	293	45.7	9.6	293	47.6	9.6	288	49.5	9.6
Gender	302			257								
Females		236	78.1%		199	77.4%	200	154	77.0%	302	236	78.1%
Males		66	21.9%		58	22.6%	200	46	23.0%	302	66	21.9%
Surgery type	-	-	-	258			200			223		
Roux-en-Y gastric bypass	-	-	-		240	93.0%		184	92.0%		207	92.8%
Sleeve gastrectomy					18	7.0%		16	8.0%		16	7.2%
Higher education	306	84	27.5%	241	73	30.3%	189	53	28.0%	204	64	31.4%
Being employed	306	193	69.4%	257	178	69.3%	198	130	65.7%	220	138	62.7%
Married/have a partner	306	202	66.2%	254	181	71.3%	199	142	71.4%	223	163	73.1%
Weight	278	130.5	22.5	271	90.0	19.1	205	92.2	19.2	223	95.7	20.0
BMI, kg/m^2^	274	43.3	5.8	252	30.9	5.7	190	31.7	5.8	206	32.9	5.7
Under/normal weight		0	0%	252	29	11.5%	190	14	7.4%	204	11	5.4%
Overweight		0	0%	252	99	39.3%	190	72	37.9%	204	48	23.5%
Obese grade 1		10	3.6%	252	69	27.4%	190	57	30.0%	204	84	41.2%
Obese grade 2		73	26.6%	252	37	14.7%	190	27	14.2%	204	40	19.6%
Obese grade 3		191	69.7%	252	18	7.1%	190	20	10.6%	204	21	10.3%
Percent weight loss (%TWL)	-	-	-	218	29.1%	9.3–53.6%*	184	26.8	5.8–53.6%*	202	23.3	− 2.3–52.0%*
> 20%	-	-	-	218	190	87.2%	184	141	76.6%	202	123	60.9%
Percent weight recurrence	-	-	-	-	-	-	190	14.7	0–70.8%*	196	25.6	0–117.3*
> 20%	-	-	-	-	-	-	190	58	30.5%	196	103	52.6%
Action planning	-	-	-	255	2.9	0.7	197	2.9	0.6	79	2.8	0.7
Coping planning	-	-	-	255	2.6	0.7	197	2.6	0.7	79	2.4	0.7
Action control	-	-	-	256	2.9	0.6	197	2.8	0.6	81	2.9	0.5

Numbers are based on all data available per time-point. Higher education: University, college, or the equivalent (education exceeding 12 years). y: year. BMI; body mass index. Sd: standard deviation. BMI categories: under/normal weight: < 25, overweight: 25–29.9, obese grade 1: 30–34.9, Obese grade 2: 35–39.9, obesity grade 3: > 40. %TWL: percent total weight loss. Likert scale for Action planning, Coping planning, and Action control ranges from 1–4

^*^Variance is presented as minimum and maximum percentage weight loss. Percent weight recurrence: weight recurrence as percent of maximum weight loss

Action-and coping planning, and action control, correlated with MVPA 1-year and 5-years after surgery (See Supplementary Table 3, Additional File 4).

### Change in action control and action and coping planning over time

**Fig. 3:**
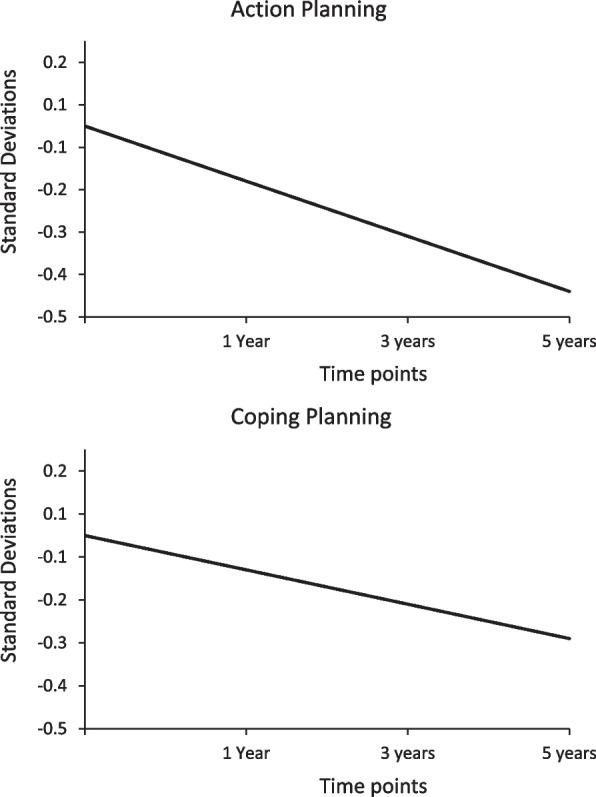
Development of Action- and Coping Planning Across 1–5 Years Post-Surgery as Measured in Standard Deviations

Table 2 shows the estimated initial mean levels (i.e., Mean (i)) and the mean-level changes, or slopes (i.e., Mean (s)), for the three self-regulation subscales, action control, action planning, and coping planning. The variability in participants’ average intercept levels 1-year after surgery (i.e., the Variance (i)) for all the three subscales indicated that individuals differed significantly in these constructs at this time-point. There was no significant change nor variability in the mean-levels of action control over the three time-points, indicating that the low levels of action control remained rather stable across time-points for all participants. However, as indicated by the slope estimates of the model (i.e., Mean (s)), the mean levels of action- and coping planning changed with − 0.13 and − 0.08 standard deviations (sd) per year, respectively, indicating that individuals' action and coping planning levels decreased on average by − 0.18 sd for each year, and thereby decreasing with as much as 0.52 and 0.30 sd, respectively, across the entire 4-year time span (Fig. 3). The decline (> 0.50 sd) indicated a moderate effect size. This decrease over time was significant and similar across all participants (Table 2).

**Table 2:** Development of the three self-regulation subscales across 1–5-years after surgery using growth curve analyses

Model	Mean (i)	Variance (i)	Mean (s)	Variance (s)	*r* (i s)	*χ*^2^	*df*	RMSEA	CFI	TLI
Action control	.00	.52^**^	-.027	.01	-.07^*^	56.03	49	.023	.99	.99
Action planning	.00	.49^**^	-.128^**^	.01	-.02	172.03	87	.060	.96	.96
Coping planning	.00	.59^**^	-.080^*^	.03	-.05	146.08	52	.082	.95	.93

i = intercept, s = slope, r (i s) = correlation between i and s, Root Mean Square Error of Approximation (RMSEA), Comparative fit index (CFI), Tucker-Lewis Index (TLI), * *p* < 0.05 ** *p* < 0.01

### Longitudinal direct, indirect, and bidirectional associations between self-regulation subscales and MVPA over time

Results from the prospective path analyses for each of the subscales showed no significant direct associations from any of the growth parameters (i.e., intercepts and slopes) to the objective measure of daily minutes in MVPA at 5-years after surgery. However, in each of the models, the MVPA 5-years after surgery was significantly predicted by MVPA at 1-year (β = 0.34, *p* = 0.006). Furthermore, MVPA at 1-year was significantly and positively associated with the intercept of action control (β = 0.36, *p* < 0.001), the intercept of action planning (β = 0.31, *p* < 0.011), and the intercept of coping planning (β = 0.32, *p* < 0.001), indicating that higher levels of action control, and action- and coping planning 1-year after surgery were associated with more daily MVPA at 1-year. MVPA at 1-year was also significantly and negatively associated with being in a relationship/being married in all the univariate models (β = − 0.24, *p* = 0.004), thus married participants were less active at 5-years.

In the model where all growth curve models for all three subscales were estimated simultaneously in one model, only the intercept of action control (i1) was significantly associated with MVPA at 1-year (β = 0.68, *p* = 0.030) when controlling for the effects of the intercepts of action- and coping planning. This indicates that among all the subscale growth factors, higher levels of action control at 1-year after surgery seems to be most strongly associated with MVPA at 1-year after surgery.

Furthermore, results from the indirect path analyses for each of the three subscales showed that the level of action control at 1-year after surgery (i.e., the intercept) was the only self-regulation construct that was indirectly associated with MVPA at 5-years after surgery through its impact on MVPA at 1-year after surgery (indirect effect β = 0.12, *p* = 0.043, 95% CI [0.004, 0.236]). No significant indirect path was found for action planning or coping planning, nor when all the three subscales and their growth factors were entered simultaneously in one indirect effects model. With regards to tests of bidirectional associations, the objective measure of daily MVPA 1-year after surgery was not significantly predictive of the development (i.e., the slope) of any of the subscales over time.

## Discussion

The current study aimed to explore how specific aspects of individuals’ self-regulation developed from 1- to 5-years after MBS, and how this development would be prospectively and bidirectionally associated with MVPA across time-points.

Results showed that: 1) On average, participants reported low levels of PA-specific self-regulation at 1-year post-surgery. 2) Action- and coping planning decreased over time whereas action control remained stable. 3) At 1-year post-surgery, higher MVPA levels were associated with higher levels of each self-regulation construct. 4) Indirect path analyses for each construct showed that only higher starting levels of action control at 1-year was indirectly associated with higher MVPA level at 5-years through its impact on MVPA at 1-year, which indicates that, of the different self-regulation constructs, action control appears to be most strongly associated with MVPA. 5) While all self-regulation constructs predicted MVPA at 1-year, and action control predicted MVPA at 5-years through its impact on MPVA at 1-year, MVPA did not predict changes in self-regulation over time, suggesting a lack of bidirectionality between the variables.

The significant individual variability between participants in initial levels of self-regulation at 1-year after MBS suggests that participants had different preconditions and capacities for becoming more physically active. Although one might assume that pre-surgery information about the necessary lifestyle changes after surgery and experiencing going through surgery, should help the patient to plan and also take more action towards being physically active [55], this appears not to apply for all patients.

The lack of individual variability in how average PA-specific action and coping planning decreased over time, suggests that patients on average will, regardless of their starting point, experience diminished ability to act when needed to meet MVPA goals over time. Average low scores at 1-year after surgery further declined through 3- and 5-years, suggesting a form of long-term ego depletion [56, 57]. The level of decline indicated a moderate effect size, suggesting many participants faced growing difficulties in regulating thoughts, emotions, and behaviors, potentially increasing psychological distress or maladaptive behaviors. An explanation for this could be that most patients are still in the typical active weight loss period at 1-year [58] which may naturally reinforce confidence, intention and specifically planning to be active [58]. However, beyond this period, these factors may be undermined with recurrence of weight gain, resulting in lower motivation to set goals, make plans, and the effort it takes to see them through. For example, weight recurrence after behavioral weight loss interventions is associated with lifestyle-related goal disengagement [59–61].

Additionally, studies show that patients are likely to be affected by unrealistic expectations related to surgery outcomes, how easy lifestyle changes will be, and the level of effort they need to invest to manage change over time [62, 63]. This makes them less prepared for everyday life when lifestyle change maintenance changes from the more automatic physiological control from surgery, to more cognitive control [64].

A novel aspect of the current study pertains to the investigation of longitudinal, indirect, and bidirectional associations between self-regulation and MVPA. In accordance with previous studies, we found a positive association between higher self-regulation and higher levels of MVPA 1-year after surgery [7, 32, 38–40]. This indicates that 1-year after surgery, patients’ ability to plan and act to be physically active are important for achieving higher MVPA levels. However, our longitudinal findings showed that MVPA 1-year after surgery was not significantly predictive of the development of any of the self-regulation constructs over time. This finding suggests that enhanced self-regulation promotes higher MVPA to a greater extent, but not necessarily the other way around.

Interestingly, we did not find any direct prospective association between planning and action control at 1-year and MVPA 5-years after surgery. However, results from the indirect path analyses suggested that action control, at 1-year indirectly predicted MVPA at 5-years through its concurrent effect on MVPA at 1-year. This result further strengthens the preliminary evidence that action control appears to be a key factor with regards to maintenance of lifestyle change in MBS patients [32, 35, 36, 39, 40]. Together, these findings further imply that evaluation of action control and interventions to strengthen action control is especially important the first year after surgery as this is the time-point in which level of self-regulation seems to influence MVPA habits the most.

At most centers performing MBS in Norway, patients receive courses and consultations in lifestyle change prior to surgery. Although these courses are based on the understanding that patients need to be prepared for how surgery affects everyday life, and how lifestyle changes are required for most optimal results [65, 66], postoperative interventions targeting behavior change may be more effective when compared to pre-surgery interventions [67, 68]. Importantly, effects of such interventions depend on participants’ compliance to attend suggested follow-ups.

Although guidelines for follow-up of MBS patients vary across institutions and between countries [69, 70], they generally focus on blood sampling, weighing, comorbidity, and dietary supplementation [70], whereas MVPA is not specifically being mentioned. More recent international guidelines on follow-up protocols post-MBS advise personally tailored pre- and post-operative follow-up with an interdisciplinary approach, including focusing on MVPA [71, 72]. In a US study, MBS patients reported lack of or inadequate advice regarding MVPA, at their bariatric facility [73]. The current follow-up system may thus not provide patients with the resources and strategies they need to achieve sustainable changes in MVPA and other health behaviors, at the time they need it the most. Guidance on MVPA during follow-up consultations could improve long-term outcome after MBS, as could also structured MVPA follow-up programs for some patients [74].

### Strengths and limitations

Strengths include the assessment of PA-specific self-regulation rarely studied in non-MBS samples and not studied in the MBS population, the objective assessment of MVPA, and the 5-year follow-up design. Also, the use of latent variables provides “cleaner” estimates, and the complex statistical methodology to examine the concurrent and longitudinal associations between the variables, needs mentioning. When interpreting the data, important limitations need consideration. No MVPA data were collected at 3-years follow-up. Participant characteristics were different between those who were retained and those who dropped out between 1- and 5-years after surgery. Accordingly, we found that participants who dropped out from 1- to 5-years MVPA assessment follow-up, where significantly heavier compared to those who participated at both time-points. Also, a higher percentage of participants who participated in the 5-years follow-up returned valid ActiGraph data compared to dropouts from the 1-year follow-up, indicating a selection bias. Further, 5-years accelerometer data was on average collected 7.88 years post-surgery, while self-regulation measures met the planned 5-year data collection. This temporal mismatch could weaken the accuracy of observed associations, as changes in self-regulation and MVPA over time may not be captured simultaneously. Readers should therefore interpret our results with caution. Bouted MVPA is often used as a proxy for structured and planned MVPA and might be more related to self-regulation than total minutes in MPVA, which was used in our study due to low accumulation of bouted MVPA minutes in our sample. Although Roux-en-Y gastric bypass is a common procedure, generalization to other types of MBS should be done with caution. As in other studies with MBS samples, the gender distribution makes our findings not generalizable to male participants alone. Data collection coincided with the COVID- 19 pandemic. The restrictions and social isolation during this period are associated with decreased PA and increased sedentary behavior [75]. During the study timeframe (2011–2020), MVPA behavior might have been increasingly impacted by changes in PA guidelines, wearable devices suggested to help improve lifestyle behaviors such as movement patterns [76], and the growing recognition of pharmacological support as an adjunct to surgical interventions [77, 78]. These changes could have particularly impacted the behaviors of individuals recovering from bariatric surgery, potentially altering their typical activity patterns and overall health outcomes. This context should be considered when interpreting the results, as the pandemic likely introduced additional variability in movement behaviors that were beyond the scope of the study's control.

### Implications

Due to the well-known negative effects of low MVPA level on long-term overall health [19, 20, 79], enhancing self-regulation is important for the best possible outcome of MBS and overall health of the patient. Assessing self-regulation, not only before and right after, but also as a part of long-term follow-up, might capture negative trends in patients’ MVPA levels. It may make it possible to act at an early stage to make it less hard to reverse negative trends and to establish good PA habits. To our knowledge, there are no existing routines guiding patients in how to improve their self-regulation abilities. This underlines a need for individualized follow-up routines specifically aimed towards how to improve MVPA levels [80]. Early post-surgery interventions aiming to optimize self-regulation skills may reduce the risk for the observed decline over time. However, our findings of a significant variation in participants self-regulation at 1-year after surgery, indicate a need to individualize follow-up, also recommended in previous literature [80]. Since action control is the recurring self-regulation construct most strongly associated with MVPA after surgery [36, 39, 40], we believe that interventions are likely to benefit from focusing on this aspect of self-regulation especially. Due to the well-known issue of dropout, additional research is also needed to identify predictors of attrition.

## Conclusions

Patients showed low levels of self-regulation 1-year after MBS. While action and coping planning decreased over time, action control remained stable. Self-regulation at 1-year after surgery was positively associated with MVPA at 1-year, with indications of action control being the self-control construct most strongly associated with MVPA. While action control at 1-year indirectly predicted MVPA at 5-years after surgery, MVPA did not predict change in self-regulation over time. Individualized interventions short-term after surgery, with long-term follow-up, focusing on promoting action control, should be further explored as a means of improving MVPA adherence and safeguarding the best possible outcome and overall health for patients after MBS.

## Supplementary Information

Supplementary Material 1.

Supplementary Material 2.

Supplementary Material 3.

Supplementary Material 4.

## Data Availability

Data will be made available on reasonable requests.

## Abbreviations

PA: Physical activity
MVPA: Moderate to vigorous physical activity
MBS: Metabolic bariatric surgery
SD: Standard deviations
CI: Confidence Interval
OBSS: Oslo bariatric surgery study
%TWL: Percent total weight loss
BMI: Body mass index (kg/m2)
REK: Regional Committees for Medical and Health Research Ethics
SEM: Structural equation modeling
CFA: Confirmatory factor analyses
CFI: Comparative fit index
RMSEA: Root mean square error of approximation
TLI: Tucker-Lewis index
SRMR: Standardized root mean square residual
