# Supplementary material for: Longitudinal associations between self-regulation and physical activity behavior following metabolic bariatric surgery; an exploratory study
Source: Int J Behav Nutr Phys Act. 2025 Apr 8;22:40. doi: 10.1186/s12966-025-01739-2 (PMC11980339; doi:10.1186/s12966-025-01739-2)
Supplement: Supplementary file 1 — Supplementary Material 1. [file 12966_2025_1739_MOESM1_ESM.docx]

**Action control – Physical activity (**[**Sniehotta, Scholz, & Schwarzer, 2005**](file:///M:\pc\Dokumenter\Prosjekt\Fedmekirurgi\Skalaer%20og%20spørreskjemaer\Spørreskjema%20Action%20control.docx#_ENREF_1)**)**

**The exercise recommendations after surgery are to be physical active enough that you sweat and breath heavily for at least 30 minutes every day (or 3,5 hours per week).**

| **During the last four weeks, I have…**  *(tick once for each question)* | 1  **Strongly Disagree** | 2  **Disagree** | 3  **Agree** | 4  **Strongly Agree** |
| --- | --- | --- | --- | --- |
| . . . constantly monitored myself whether I am physical active as recommended |  |  |  |  |
| .. watched carefully that I have followed the recommendations for physical activity |  |  |  |  |
| ..often thought about my intentions about being physical active |  |  |  |  |
| .. always been aware that I should be physical active |  |  |  |  |
| .. really tried to be physical active on a regular basis |  |  |  |  |
| .. tried my best to be physical active as recommended |  |  |  |  |
|  |  |  |  |  |

**Action + Coping planning - Physical activity (Sniehotta, 2009)**

| **I already have concrete plans for...**  (tick once for each question) | | **1**  **Not at all** | **2**  **Barely** | **3**  **Mostly** | **4**  **Exactly** |
| --- | --- | --- | --- | --- | --- |
| ...when to be physical active | |  |  |  |  |
| ...where to be physical active | |  |  |  |  |
| ...how to be physical active | |  |  |  |  |
| ...how often to be physical active | |  |  |  |  |
| ...with whom to be physical active | |  |  |  |  |
| ...what to do if something intervenes |  |  |  |  |  |
| ...what to do if I miss an exercise session |  |  |  |  |  |
| …what to do in difficult situations in order to stick to my intentions |  |  |  |  |  |
| …when to especially watch out in order to stay committed to my plans |  |  |  |  |  |

Sniehotta FF, Scholz U, Schwarzer R. Bridging the intention–behaviour gap: Planning, self-efficacy, and action control in the adoption and maintenance of physical exercise. Psychol Health. 2005;20(2):143-160.

Sniehotta FF. Towards a theory of intentional behaviour change: Plans, planning, and self-regulation. Br J Health Psychol. 2009;14(2):261-273.
