# Supplementary material for: Longitudinal associations between self-regulation and physical activity behavior following metabolic bariatric surgery; an exploratory study
Source: Int J Behav Nutr Phys Act. 2025 Apr 8;22:40. doi: 10.1186/s12966-025-01739-2 (PMC11980339; doi:10.1186/s12966-025-01739-2)
Supplement: Supplementary file 2 — Supplementary Material 2. [file 12966_2025_1739_MOESM2_ESM.docx]

**Supplementary Table 1.** Model fit of self-regulation subscales and test of measurement invariance over time.

| Model | #items(modind) | χ^2^(df) | RMSEA [95%CI] | CFI | TLI | SRMR | ∆ χ^2^ | ∆df | *p* |
| --- | --- | --- | --- | --- | --- | --- | --- | --- | --- |
| CFA per subscale |  |  |  |  |  |  |  |  |  |
| 1. Action control 1-y^1^ | 4 (1&2) | 0.128 (1) | .000 [.000, .118] | 1.0 | 1.0 | .001 |  |  |  |
| 2. Action control 3-y | 4 (1&2) | 0.159 (1) | .000 [.000, .140] | 1.0 | 1.0 | .002 |  |  |  |
| 3. Action control 5-y | 4 (1&2) | 0.364 (1) | .000 [.000, .247] | 1.0 | 1.0 | .005 |  |  |  |
| *Invariance testing over time* |  |  |  |  |  |  |  |  |  |
| Configural invariance | (5&6) | 29.359 (36) | .000 [.000, .031] | 1.0 | 1.0 | .045 |  |  |  |
| Weak invariance | (5&6) | 36.706 (42) | .000 [.000, .033] | 1.0 | 1.0 | .061 | 7.347 | 6 | >.01 |
| Strong invariance | (5&6) | 51.086 (48) | .015 [.000, .044] | .99 | .99 | .069 | 14.38 | 6 | >.01 |
| 1. Action planning 1-y | 5 | 6.653 (5) | .036 [.000, .100] | .99 | .99 | .010 |  |  |  |
| 2. Action planning 3-y | 5 (2&4) | 15.786 (4) | .122 [.063, .188] | .99 | .96 | .023 |  |  |  |
| 3. Action planning 5-y | 5 | 14.545 (5) | .155 [.066, .251] | .97 | .95 | .024 |  |  |  |
| *Invariance testing over time* |  |  |  |  |  |  |  |  |  |
| Configural invariance |  | 161.088 (72) | .068 [.054, .082] | .96 | .95 | .054 |  |  |  |
| Weak invariance |  | 168.199 (80) | .064 [.051, .078] | .96 | .95 | .066 |  |  |  |
| Strong invariance |  | 172.017 (.86) | .061 [.048, .074] | .96 | .96 | .065 |  |  |  |
| 1. Coping planning 1-y | 4 (1&2) | .006 (1) | .000 [.000, .044] | 1.0 | 1.0 | .000 |  |  |  |
| 2. Coping planning 3-y | 4 (1&2) | 2.671 (1) | .092 [.000, .234] | .99 | .98 | .005 |  |  |  |
| 3. Coping planning 5-y | 4 (1&2) | 4.047 (2) | .114 [.000, .276] | .99 | .98 | .012 |  |  |  |
| Configural invariance |  | 135.807 (39) | .096 [.079, .114] | .95 | .91 | .047 |  |  |  |
| Weak invariance |  | 143.250 (45) | .090 [.074, .107] | .95 | .92 | .061 |  |  |  |
| Strong invariance |  | 145.330 (51) | .083 [.067, .099] | .95 | .93 | .062 |  |  |  |

Note. * = modification indices residual correlations coping planning and action planning, ^1^ = removed items 2 and 3 due to poor reliability/factor loading. y: year. CFA: Confirmatory Factor Analyses. RMSEA: Root Mean Square Error of Approximation. CFI: Comparative Fit Index. SRMR: Standardized Root Mean Square Residual.
