# Supplementary material for: Longitudinal associations between self-regulation and physical activity behavior following metabolic bariatric surgery; an exploratory study
Source: Int J Behav Nutr Phys Act. 2025 Apr 8;22:40. doi: 10.1186/s12966-025-01739-2 (PMC11980339; doi:10.1186/s12966-025-01739-2)
Supplement: Supplementary file 3 — Supplementary Material 3. [file 12966_2025_1739_MOESM3_ESM.docx]

Supplementary Table 2. Description of participants in the Oslo Bariatric Surgery Study at each assessment time-point after surgery

|  | Pre-surgery | | | 1-y | | | 3-y | | | 5-y | | |
| --- | --- | --- | --- | --- | --- | --- | --- | --- | --- | --- | --- | --- |
|  | N | Mean/  sum | Sd/% | N | Mean/  sum | Sd/% | N | Mean/  sum | Sd/% | N | Mean/  sum | Sd/% |
|  |  |  |  |  |  |  |  |  |  |  |  |  |
| Age | 112 | 44.8 | 9.4 | 106 | 45.9 | 9.4 | 89 | 49.0 | 8.7 | 112 | 54.3 | 9.4 |
| Gender |  |  |  |  |  |  |  |  |  |  |  |  |
| Females | 112 | 90 | 80.4% | 112 | 90 | 80.4% | 112 | 74 | 78.7% | 112 | 90 | 80.4% |
| Males |  | 22 | 19.6% |  | 22 | 19.6% |  | 20 | 21.3% |  | 22 | 19.6% |
| Surgery type | - | - | - | 112 |  |  | 112 |  |  | 112 |  |  |
| Roux-en-Y gastric bypass | - | - | - |  | 111 | 99.1% |  | 111 | 99.1% |  | 111 | 99.1% |
| Sleeve gastrectomy | - | - | - |  | 1 | 0.1% |  | 1 | 0.1% |  | 1 | 0.1% |
| Higher education | 111 | 38 | 34.2% | - | - | - | - | - | - | - | - | - |
| Being employed | 106 | 87 | 82.1% | 112 | 81 | 72.3% | 93 | 67 | 72% | 96 | 66 | 68.8% |
| Married/have a partner | 112 | 68 | 61.3% | 111 | 73 | 65.8% | 93 | 64 | 68.8% | 98 | 75 | 76.5% |
| Weight | 109 | 124.9 | 19.5 | 105 | 89.1 | 17.3 | 91 | 90.4 | 17.,2 | 103 | 94.6 | 18.1 |
| BMI, kg/m^2^ | 109 | 42.9 | 5.1 | 105 | 30.6 | 5.0 | 91 | 31.0 | 4.8 | 102 | 32.7 | 5.2 |
| Under/normal weight |  | 0 | 0% |  | 11 | 10.6% |  | 7 | 7.7% |  | 4 | 3.9% |
| Overweight |  | 0 | 0% |  | 46 | 44.2% |  | 37 | 40.7% |  | 24 | 23.5% |
| Obese grade 1 |  | 5 | 4.6% |  | 22 | 21.2% |  | 28 | 30.8% |  | 43 | 42.2% |
| Obese grade 2 |  | 27 | 24.8% |  | 20 | 19.2% |  | 14 | 15.4% |  | 20 | 19.6% |
| Obese grade 3 |  | 77 | 70.7% |  | 5 | 4.8% |  | 5 | 5.5% |  | 11 | 10.8% |
| Percent weight loss (%TWL) | - | - | - | 102 | 28.9% | 9.5-42.9% | 88 | 27.3% | 5.8-44.9% | 100 | 23.1% | 0.8-43.2% |
| >20% | - | - | - |  | 91 | 89.2% |  | 69 | 78.4% |  | 62 | 62.0% |
| Percent weight recurrence | - | - | - | - | - | - | 89 | 13.9% | 0-65.2% | 93 | 25.9% | 0-96.8% |
| >20% | - | - | - | - | - | - |  | 27% | 30.3% |  | 46 | 49.5% |
| Action planning | - | - | - | 111 | 3.1 | 0.7 | 93 | 3.0 | 0.7 | 79 | 2.8 | 0.7 |
| Coping planning | - | - | - | 111 | 2.7 | 0.7 | 93 | 2.7 | 0.7 | 79 | 2.4 | 0.7 |
| Action control | - | - | - | 112 | 3.0 | 0.7 | 93 | 2.9 | 0.6 | 81 | 2.9 | 0.5 |

Note. Numbers are based on participants who returned valid ActiGraph data at 1-year (1-y) after surgery and were eligible to take part in the 5-year ActiGraph follow-up (5-y). Higher education: University, college, or the equivalent (education exceeding 12 years). y: year. BMI; body mass index. Sd: standard deviation. BMI categories: under/normal weight: <25, overweight: 25-29.9, obese grade 1: 30-34.9, Obese grade 2: 35-39.9, obesity grade 3: >40. %TWL: percent total weight loss. Likert scale for Action planning, Coping planning, and Action control ranges from 1-4. *Variance is presented as minimum and maximum percentage weight loss. Percent weight recurrence: weight recurrence as percent of maximum weight loss.
