# Supplementary material for: Longitudinal associations between self-regulation and physical activity behavior following metabolic bariatric surgery; an exploratory study
Source: Int J Behav Nutr Phys Act. 2025 Apr 8;22:40. doi: 10.1186/s12966-025-01739-2 (PMC11980339; doi:10.1186/s12966-025-01739-2)
Supplement: Supplementary file 4 — Supplementary Material 4. [file 12966_2025_1739_MOESM4_ESM.docx]

**Supplementary Table 3.** Correlation between self-regulation and moderate-to vigorous physical activity at each measuring time-point.

|  | **MVPA 1-y** |  |  | | **MVPA 5-y** |  |  | |
| --- | --- | --- | --- | --- | --- | --- | --- | --- |
|  | *r* | *p* | CI95% | | *r* | *p* | CI95% | |
| Action planning 1-y | .29 | **.002** | .11 | .45 | .18 | .124 | -.05 | .38 |
| Coping planning 1-y | .25 | **.007** | .07 | .42 | .14 | .206 | -.08 | .35 |
| Action control 1-y | .34 | **<.001** | .17 | .50 | .26 | **.022** | .04 | .45 |
| Action planning 3-y | .21 | **.048** | .00 | .39 | .20 | .101 | -.04 | .41 |
| Coping planning 3-y | .18 | .086 | -.03 | .37 | .14 | .254 | -.10 | .36 |
| Action control 3-y | .31 | .**003** | .11 | .48 | .10 | .406 | -.14 | .33 |
| Action planning 5-y | .18 | .119 | -.05 | .38 | .28 | **.013** | .06 | .48 |
| Coping planning 5-y | -.01 | .921 | -.23 | .21 | .24 | **.037** | .02 | .44 |
| Action control 5-y | .21 | .055 | -.00 | .41 | .27 | **.017** | .05 | .46 |

Note. MVPA: Moderate-to vigorous physical activity. y: years. CI: Confidence Interval.
